# *MMHelper*: An automated framework for the analysis of microscopy images acquired with the mother machine

**DOI:** 10.1038/s41598-019-46567-0

**Published:** 2019-07-12

**Authors:** Ashley Smith, Jeremy Metz, Stefano Pagliara

**Affiliations:** 1 Living Systems Institute, University of Exeter, Exeter, United Kingdom; 2 Biosciences, University of Exeter, Exeter, United Kingdom

**Keywords:** Lab-on-a-chip, Biological fluorescence, Bacterial techniques and applications, Imaging techniques

## Abstract

Live-cell imaging in microfluidic devices now allows the investigation of cellular heterogeneity within microbial populations. In particular, the mother machine technology developed by Wang *et al*. has been widely employed to investigate single-cell physiological parameters including gene expression, growth rate, mutagenesis, and response to antibiotics. One of the advantages of the mother machine technology is the ability to generate vast amounts of images; however, the time consuming analysis of these images constitutes a severe bottleneck. Here we overcome this limitation by introducing *MMHelper* (10.5281/zenodo.3254394), a publicly available custom software implemented in Python which allows the automated analysis of brightfield or phase contrast, and any associated fluorescence, images of bacteria confined in the mother machine. We show that cell data extracted via *MMHelper* from tens of thousands of individual cells imaged in brightfield are consistent with results obtained via semi-automated image analysis based on ImageJ. Furthermore, we benchmark our software capability in processing phase contrast images from other laboratories against other publicly available software. We demonstrate that *MMHelper* has over 90% detection efficiency for brightfield and phase contrast images and provides a new open-source platform for the extraction of single-bacterium data, including cell length, area, and fluorescence intensity.

## Introduction

Phenotypic heterogeneity is a common feature within isogenic bacterial populations^1–3^. Cell-to-cell variations have been observed in bacterial growth rate^3^, virulence^4^, and resistance to stress^1^. As a result, it has been suggested that such heterogeneity may allow some cells to survive within fluctuating environments^1,5–8^ and hence promote evolutionary adaptation^9,10^. Traditional microbiological assays are based on ensemble measurements and thus unable to measure cell-to-cell differences within microbial populations. In contrast, microfluidics allows the precise manipulation of fluids at the submillimetre level^11^ and when used in combination with microscopy can be utilised for biological assays with single-cell resolution^12,13^. Microfluidics has already been adapted for investigating heterogeneity across multiple domains of life. For instance, Hansen *et al*. developed a protocol which enables measurement of signalling dynamics in single yeast cells^14^, Li *et al*. investigated heterogeneity in the migration ability of a population of lung cancer cells^15^, Yuan *et al*. looked at the effects of genome deletions on bacterial growth^16^, Pagliara *et al*. showed that embryonic stem cells exhibit auxetic properties^17^, and Otto *et al*. measured the mechanical deformability of single cells to identify cell sub-populations in whole blood samples^18^. There are a multitude of microfluidic designs and devices available for investigating single bacterial cells. One popular example is the mother machine^19^, which provides an ideal platform for tracking single bacterial cells over time while continuously supplying growth nutrients or compounds to be tested such as antibiotics.

Wang *et al*. designed the mother machine (MM) to allow the trapping of a single mother cell at the dead-end of each of thousands of microfluidic channels and the tracking of its daughter cells over hundreds of generations^19^. This tool has since been employed to investigate a variety of research questions with single-cell resolution. Tanouchi *et al*. and Kaiser *et al*. used the MM to investigate gene regulation^20–22^. Robert *et al*. and Uphoff investigated the emergence of mutations in single cells and the dynamics of mutagenesis^23,24^. Moolman, *et al*. utilised it to explore protein stoichiometry and dynamics^25^ whereas Chait *et al*. used it to engineer bacterial population behaviour^26^. Multiple groups have use it to investigate single cell response to antibiotics^4,27,28^, and Yang *et al*. studied bacterial adaptation under physical confinement^29^.

Some research groups have developed software which can be used for the analysis of images of bacteria confined in the mother machine^30^, although most still use scripts customised around their experimental and imaging set-up^23,26,27,31^. Initially, Arnoldini, *et al*. developed *mmj*, a semi-automatic ImageJ plug-in which facilitates the analysis of mother machine images^4^. However, it is inefficient to use this semi-automated approach on thousands of images. Sachs *et al*. developed *Molyso* an unsupervised software implemented in Python^30^. *Molyso*, provides a fast and efficient framework capable of analysing 90 GB of mother machine images in 30 min. Nonetheless, their program has limitations which prevent its use by the wider mother-machine community, including not being suitable to analyse standard brightfield images, and constraints on initial channel orientation. Another ImageJ plug in, *MoMA*, is also available and the authors claim to achieve unprecedented accuracy in segmenting and tracking bacteria^22^. However, we were unable to install and run *MoMA*, on any datasets, within a reasonable (2 hour minimum) time period. Using the suggested installation method we successfully installed *MoMA* but always encountered a FIJI exception error when trying to run the application due to its dependency on Gurobi, even when running on *MoMA*’s own image set.

In order to overcome the limitations above, we introduce *MMHelper*, an analysis framework that, to the best of our knowledge, is the first fully automated program applicable to multiple imaging modalities of the mother machine. *MMHelper* is implemented as a user-friendly python module which detects bacteria confined within the MM and tracks their progeny and fate through time. These detected bacterial regions can then be used to access information on length and area as well as any accompanying fluorescence intensity data. We demonstrate that by using MMHelper, brightfield imaging can be used for extracting phenotypic information from individual bacteria (e.g. length, width, morphology) in the mother machine as well as phase contrast imaging; with the added value that brightfield imaging does not rely on the use of specialised optical components. Furthermore, we have recently used *MMHelper* to analyse the response to antibiotics of 11,823 single bacteria thus generating novel insight on the physiology of phenotypic variants^28^. Therefore, we believe that the efficiency and accuracy of *MMHelper* will assist the investigation of a variety of biological questions by significantly improving the throughput and reliability of mother machine experiments.

## Methods

**Figure 1:**
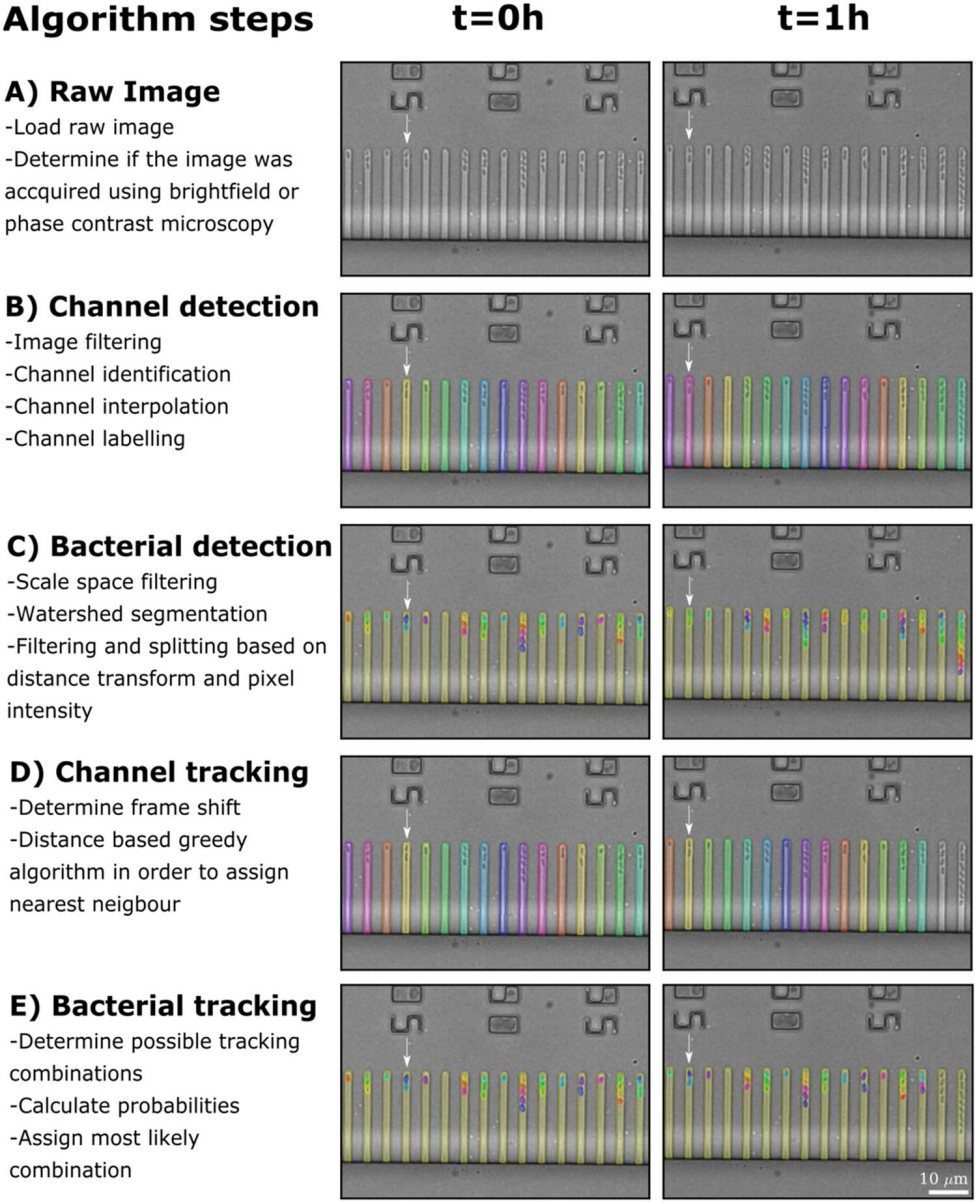
Overview of the analysis pipeline. The analysis pipeline is broken down into five major steps. **(A)** The imaging mode is detected, determining whether images are brightfield or phase contrast. **(B)** Channels are detected, assigned specific labels and ordered consecutively from left to right. **(C)** Bacteria are detected in each channel. **(D)** Channels are tracked throughout the image time-series. In these representative images, the mother machine device at t = 1 h has moved approximately 10 μm to the left with respect to t = 0 h, as indicated by the arrow. Our algorithm quantifies this frame shift and relabels each channel accordingly, for example the channel indicated by the arrow is recoloured in yellow. **(E)** After channel tracking, the detected bacteria in each channel are tracked accordingly and relabelled where necessary, each bacterium keeping the same unique colour through consecutive time points as indicated by the arrow.

**Figure 2:**
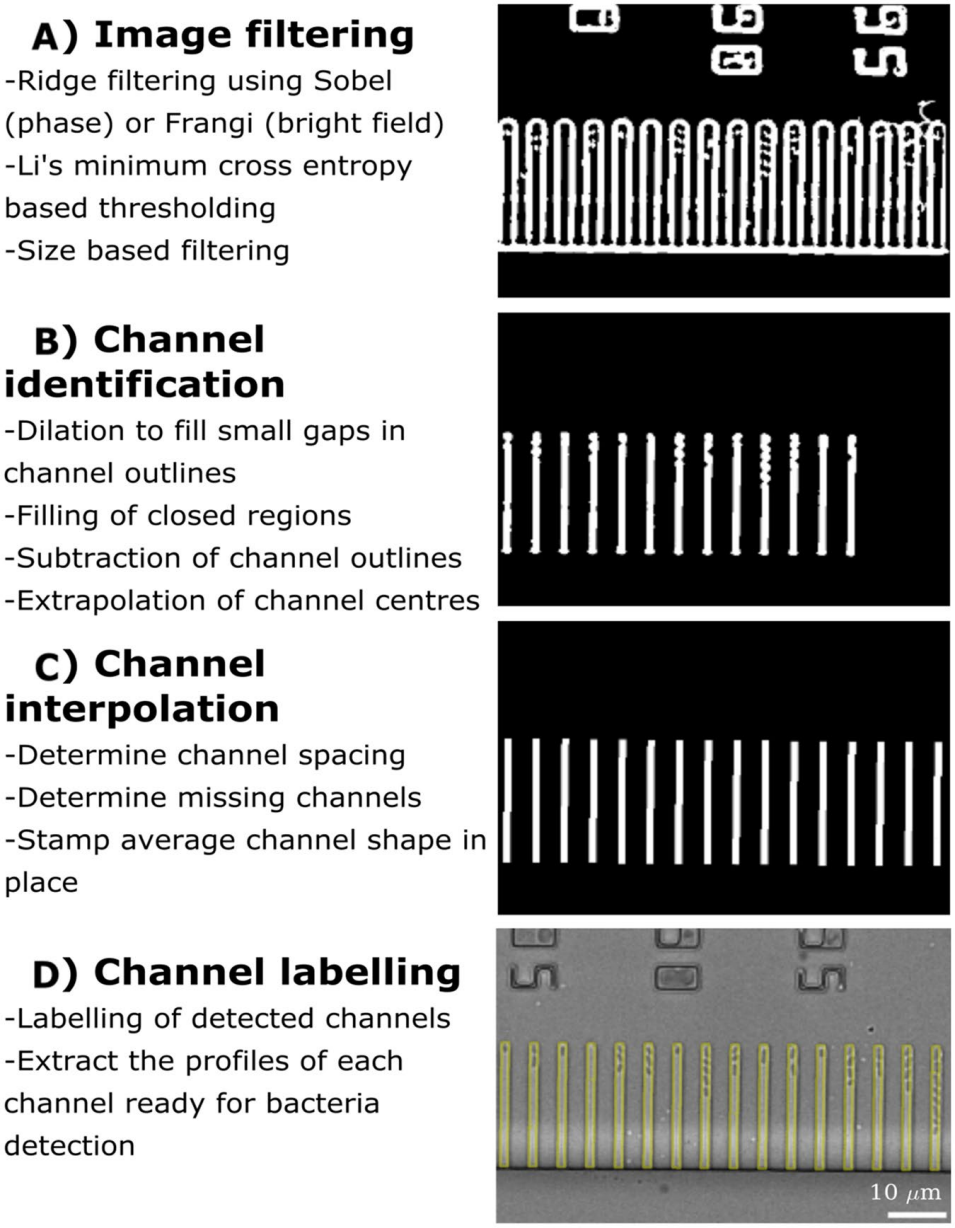
Pipeline for channel detection. (**A)** The original image is filtered (Sobel for phase images and Frangi for brightfield) followed by thresholding to identify potential ridges. These ridges are then filtered by size to leave the masks of the channels. **(B)** A new mask is created with the centre of each channel filled and through a simple subtraction of the previous mask with the new one, the centre of each channel is extrapolated. These masks can appear irregular in shape due to the presence of the bacteria they host. Consequently, new profiles are determined by creating vectors around the perimeter to form an average channel shape. **(C)** The spacing between these channels is determined and, after interpolation to determine the location of missing channels, the average channel shape is stamped in place. Noteworthy, our algorithm performs well also with images where the main channel is not horizontal resulting in slightly staggered labels. **(D)** A yellow contour is drawn around each label to delineate the detected channels.

**Figure 3:**
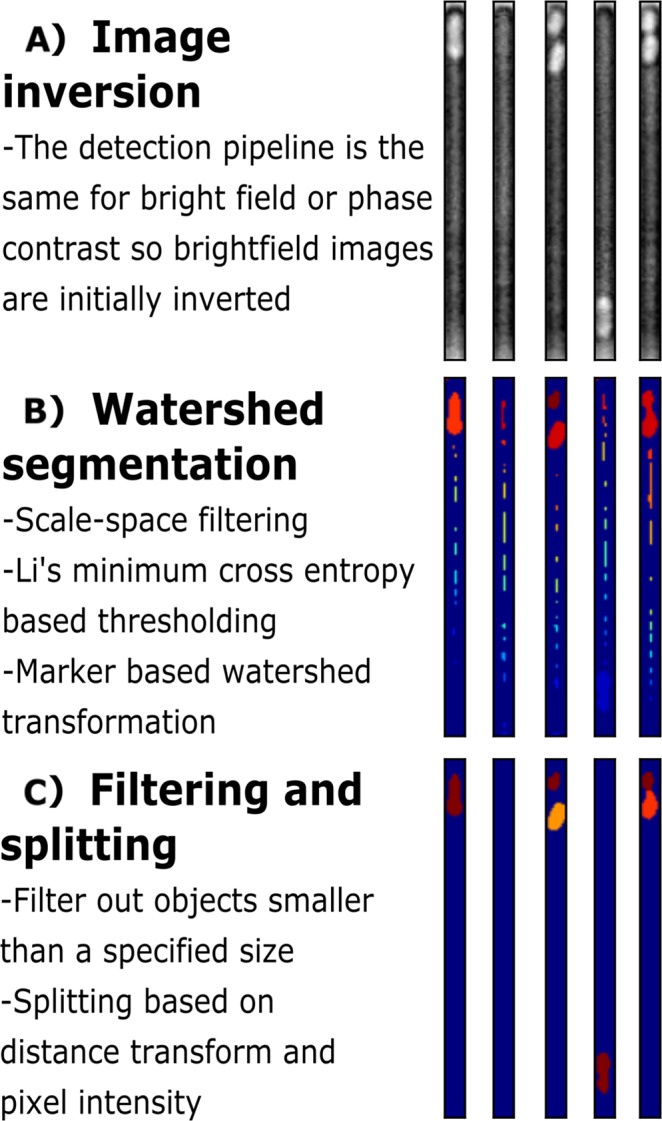
Pipeline for bacteria detection. (**A**) By using the masks for the detected channels, the corresponding original image for each channel is identified and the image inverted using background subtraction. **(B)** This is followed by scale space filtering and thresholding. As a result, markers are identified that can be used for a watershed transformation. **(C)** Each single element within each channel identified by the watershed transformation is given a unique label, represented by a different colour. The result of the watershed is filtered to remove non-bacterial particles. Bacterial splits are identified, using a combination of width and pixel intensity, and a mask of the detected bacteria produced using a combination of distance transformation and pixel intensity along the skeleton.

Our image analysis pipeline can be decomposed into two core stages, detection and tracking, which are followed by the extraction of the temporal changes of single-cell parameters including length, width, area, and fluorescence intensity. After determining the imaging modality (1A), each stage is comprised of channel-centric (Fig. 1B,D) and bacteria-centric (Fig. 1C,E) sub-stages. The detection stages (Figs 1B,C and S1) take place independently of the time-point of the experiment and are shown in more detail in Fig. 2A–D and Fig. 3A–D, respectively. In comparison, the tracking stages (Fig. 1D,E) are performed relative to the previous time point (i.e. the t = 0 h left hand panel images are used as a reference for the tracking on the t = 1 h right hand panel images).

### Data organisation and loading

Each image is loaded as a multi-dimensional numpy array using the scikit-image module. For experiments including fluorescence images, these arrays are split such that detection is only performed on the brightfield (or phase contrast) images. *MMHelper* can be run specifically on single images or on image time-series and it also contains a batch run mode. This mode allows the analysis of a whole folder that contains images from tens of different time points and areas of the MM. In this instance, a naming protocol is used to associate images with areas on the chip. Specifically, a string is used at the start followed by an underscore that identifies which MM area the respective image is from. After this underscore, a time stamp is used in order to sort the images in chronological order (e.g. a suitable filename for an image of area 1 of the MM acquired at 12:33:01 on the 16^th^ October 2017 would be: “Area01_171016_123301.tiff”).

### Detection

The first stage of the detection process is to determine whether the image is a phase or brightfield image (Fig. 1A). We noted that the pixel intensity distributions of brightfield and phase-contrast images, obtained with similar N.A. objectives, are significantly different. Therefore, we used the skewness of the pixel intensity distribution to detect the imaging modality. As we have a large sample size in terms of pixel count (for a square image that is 1000 pixels in length: ${n}_{pixels}\approx 1,{000}^{2}=1,000,000$), we used the uncorrected expression for the skewness *G*_1_^32^, with the samples third and second central moments of the pixel data, *m*_3_ and *m*_3_ respectively.

$${G}_{1}=\frac{{m}_{3}}{{{m}_{2}}^{3/2}}$$

If this equation returns *G*_1_ as a positive value, the image is assumed to be phase contrast, whereas a negative value suggests the input image was acquired in brightfield.

After determining the imaging modality, the input image is filtered (Fig. 2A) using a gradient magnitude Sobel edge for phase contrast images or Frangi ridge filter for brightfield images^33^.

The edge or ridge filtering accentuates the channel outlines, and is followed by Li’s iterative minimum cross-entropy based automated thresholding^34^ to binarize the image. This mask image is labelled using connected-component labelling, and the labelled regions are filtered based on area to remove non-channel regions.

The resulting channel-outlines are morphologically dilated to close small gaps in the outline, and the subsequent closed regions are filled using a region-filling algorithm. These inner channel regions are extracted as the difference between the outlines and the filled regions (Fig. 2B). The inner-channel perimeters are converted to pixel locations and, by determining the pixel locations that are farthest apart, channel vectors are generated. These vectors are filtered for length to select only regions in a predetermined range (default: 100–400 pixels) based on the images acquired from our typical experimental set-up, however they can be adjusted using a scale factor (see additional parameters section). The resulting vectors correspond to the long channel edges, therefore the perpendicular distance between them is also filtered to ensure that the selected channels correspond to single channels. The resulting channel regions form the basis for a subsequent interpolation stage (Fig. 2C). First, the aforementioned channel regions are analysed to determine the single channel-to-channel spacing, to allow the identification of undetected channels. Using this spacing, the positions of eventually undetected channels are interpolated from the detected channel positions. The detected average channel shape is stamped into each interpolated position. Using the channel contours, the perimeter of each detected channel can be seen in the final output images (Fig. 2D). Note that at least three channels must be detected in any given image to allow the algorithm to attempt interpolation. If two or less channels are detected the algorithm warns the user that it was unable to accurately detect channels in this image, and the frame is not considered for further detection.

The next sub-stage in detection is to detect bacteria within the channels identified from the process above. In these images, the bacteria initially appear darker than the background (Fig. 2D). Therefore, the images are inverted to allow for the use of standard algorithms to detect bright objects on dark background. To do this, the background intensity for each channel is estimated using a rolling ball filter and subtracted from its respective image^35^ (Fig. 3A). Furthermore, by subtracting the background intensity, the watershed segmentation can remain the same for bacteria located anywhere along the channel profile (Fig. S2).

These channel images are then processed as follows: first each channel image is scale-space filtered^36^ using a Laplace of Gaussian convolution at multiple scales, and maximum-projected along the scale axis (Fig. 3B). Using these filtered channel images, a threshold value is determined using Li’s algorithm to avoid over-segmentation of empty channels. Each filtered channel image is then binarized using this threshold value and outlines generated by taking the difference between the dilation (grow) and the erosion (shrink) of the initial binary image. An initial crude region-splitting stage is included as occasionally multiple bacteria are detected as a single region, which reduces the accuracy of the region size filtering step. For this, the algorithm uses the marker-controlled Watershed transform^37^. Markers are generated from all regions greater than a predefined distance from the mask background, and used to delineate bacteria. These regions are finally filtered for width and size (Fig. 3C). Following the initial bacteria segmentation, a second dedicated bacteria-splitting stage was included to improve the segmentation quality of adjacent bacteria (Fig. 3C). The initially detected bacteria are skeletonised and “splits” identified using a combination of distance transformation and pixel intensity, with the threshold values determined using the median and median absolute deviation of all the initially detected bacteria from the original image.

### Tracking

**Figure 4:**
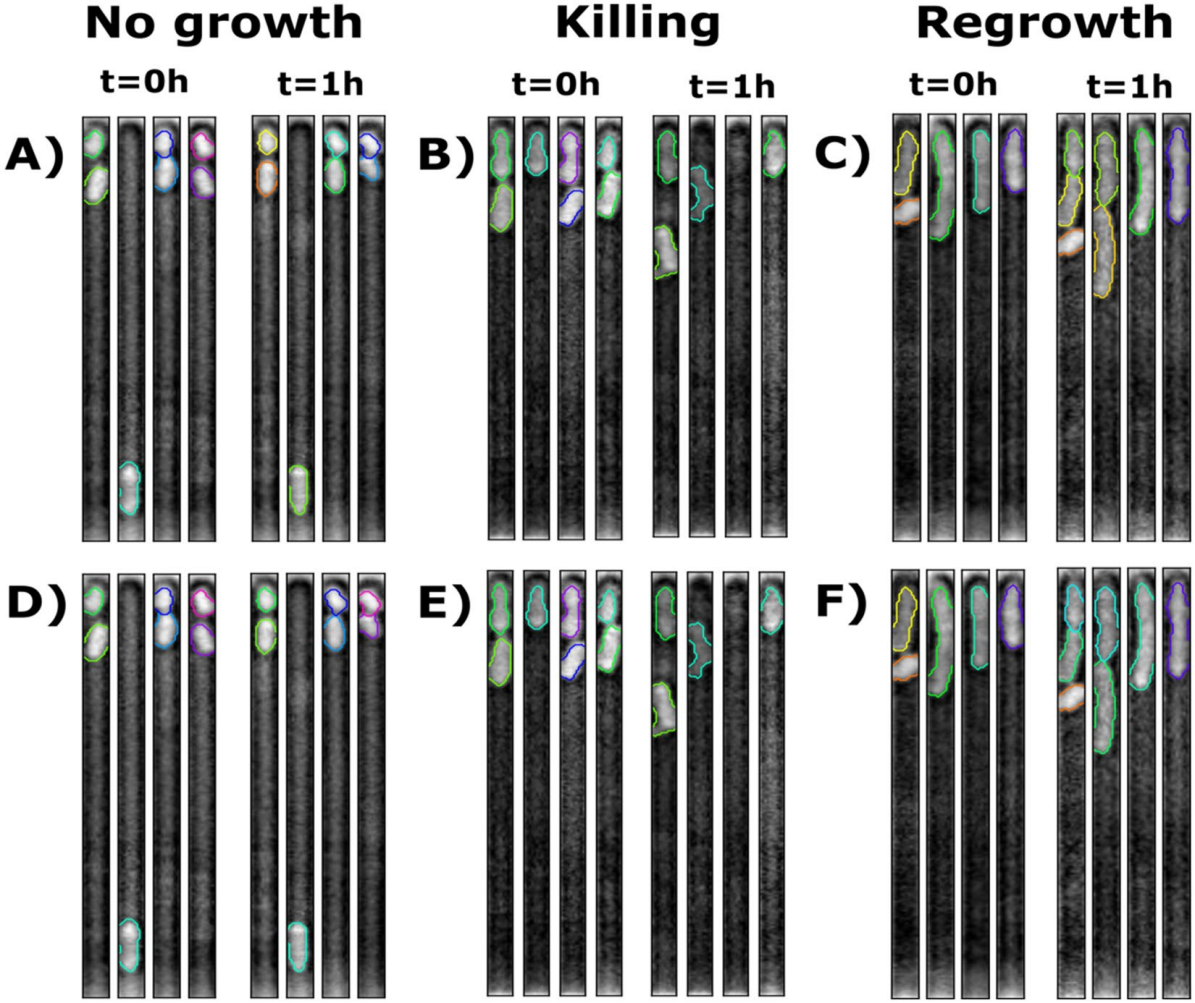
Overview of bacteria tracking. Individual bacteria detected in an experiment using **(A)** minimal medium, **(B)** antibiotic treatment, or **(C)** growth medium at t = 0 and at t = 1 h (channels at the left and right hand side of each panel, respectively). **(D–F)** Corresponding tracked bacteria are relabelled, where necessary (e.g. second channel from the left in D), at t = 1 h so that their label (i.e. contour colour) matches that at t = 0. When a division occurs each of the offspring is assigned a new unique label (e.g. first and second channel from the left in **F**).

The detected channels and bacteria are tracked in two stages. First global frame shift is determined for whole images using cross-correlation based template matching^38^. This allows channels from consecutive timepoints to be matched using simple distance-based greedy assignment, which matches each point to its nearest neighbour as long as it is also the nearest neighbour to that point. To do so, channel centroid positions are extracted and channels in consecutive frames are linked if each is the nearest neighbour to the other (Fig. 1D). Once channels have been tracked in adjacent time frames, bacteria can be tracked in each channel. This proceeds according to a simple multiple-hypothesis tracking where probabilities of all possible assignments are calculated. These assignments take into account the centroid position and area of each bacterium, as well as adjustable probabilities that each bacterium remains an individual entity (no-change, Fig. 4A), or fades away from the channel (cell death, Fig. 4B), or gives rise to progeny (cell division, Fig. 4C). These events can occur in a number of different combinations to produce the number of bacteria detected in the current frame relative to the preceding frame (t = 1 h compared to t = 0 in Fig. 4A–C). Therefore, a list of all these possible combinations is generated and for each of these possibilities the total number of bacterial divisions that would be required is determined. A probability based on the change in area between the bacteria and its offspring is determined and normalised by the number of divisions. A second probability based on the change in centroid, is calculated taking into account that for each division the change in centroid location is expected to move by half the length of an average bacterium. Finally, the algorithm calculates the likelihood of a cell dividing, lysing, or remaining a single cell between consecutive time points. All three of these probabilities are then multiplied together to determine the overall likelihood that the given event occurred for an individual bacterium. The determined probability for each bacterium within the channel is multiplied to produce an overall probability for the respective combination of events. The resulting, most probable, combination is then used to correctly relabel each bacterium in each image (e.g. second channel from the left in Fig. 4D), with newly generated bacteria assigned a new unique label (e.g. first and second channels from the left in Fig. 4E).

### Extraction of single-cell parameters

**Figure 5:**
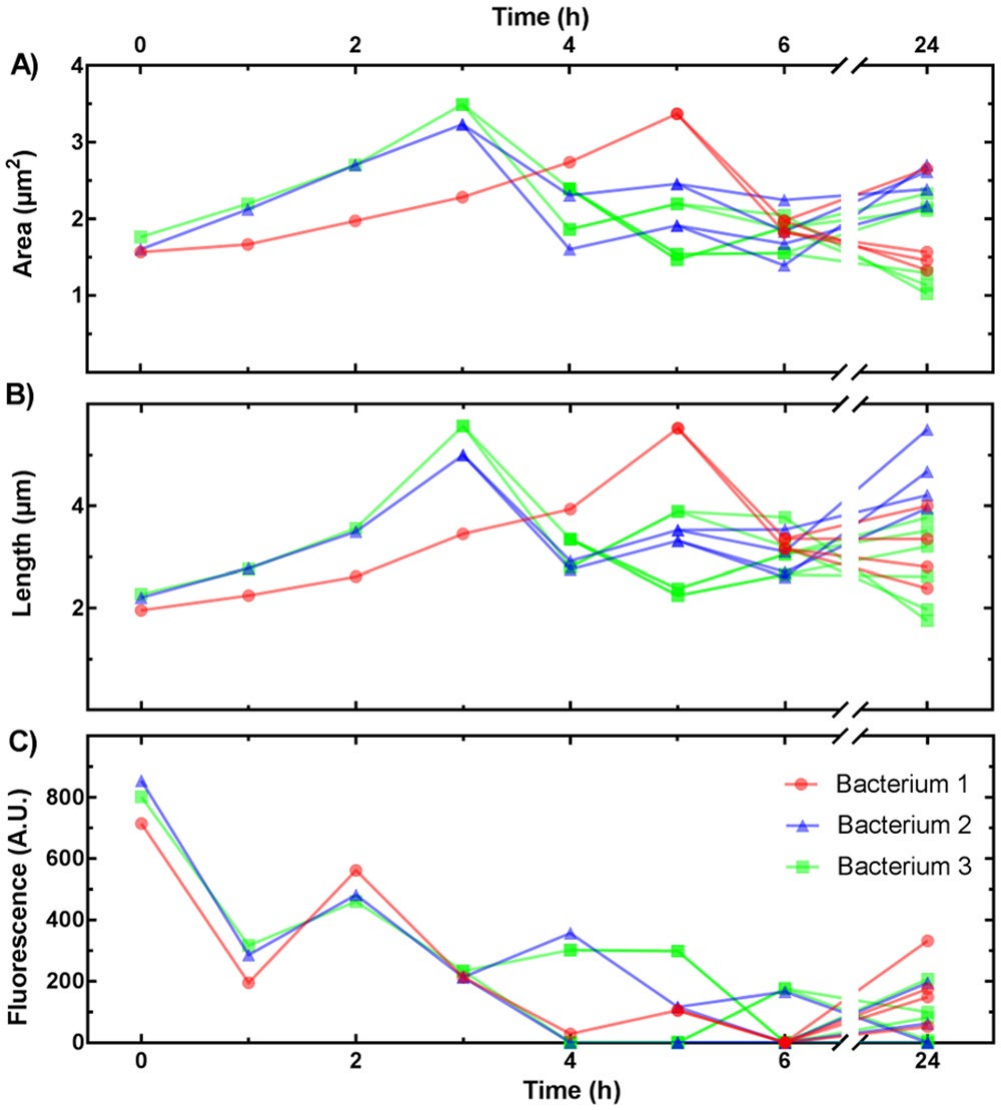
Dynamics in single-bacterium parameters. Temporal changes in **(A)** area, **(B)** length, and **(C)** GFP fluorescence for three representative bacteria, and their progeny, growing in lysogeny broth. Data bifurcations indicate bacterial divisions, e.g. bacterium 3 divided at t = 3 h and its daughters divided at t = 5 h.

Once bacteria detection and tracking has been completed, extraction of all quantities of interest can be achieved through the detected and tracked region-based properties. Each bacterium’s length, width, and area are determined using the various standardised algorithms presented via the *regionprops* function. The binary masks can then be used to extract the raw fluorescence intensity values from the corresponding fluorescence images reporting for example the activity of transcriptional reporters or the intracellular accumulation of spectrally distinct substrates. The background fluorescence is obtained from the empty areas (parts of the channels not containing detected bacteria) of each channel and subtracted from each respective bacterium’s fluorescence intensity. These quantities are then saved in a csv file. We have recently used *MMHelper* to measure the temporal changes in promoter activity in 11,823 individual *Escherichia coli*^28^. Figure 5A–C report the temporal changes in area, length and GFP fluorescence for three representative bacteria, and their progeny, growing in lysogeny broth. The fluorescence reported in Fig. 5C is the mean pixel intensity and the gradual decline in the fluorescence values reported is not due to photobleaching, but is a genuine proxy for the expression of the multi efflux pump *tolC* (the promoter upstream of GFP in the plasmid carried by the strain), due to the reduction of cellular stress upon continuous exposure to fresh media, similar to the profile we previously observed^28^.

### Additional parameters and module usage

*MMHelper* can be used to analyse image time series acquired with different microscopy setups (e.g. different objective magnification and numerical aperture, different cameras) by adjusting a single “Scale factor” parameter. Furthermore, the user can specify how many fluorescence image channels are acquired for each brightfield (or phase contrast) image. More information on parameters and how to adjust them will be available on the repository wiki page (https://github.com/jmetz/mmhelper/wiki).

Due to the 2D nature of *MMHelper’s* detection, it performs the analysis on any image orientation and there is no need for tilt correction. Furthermore, the modular nature of MMHelper makes it suitable for future adaptation to slightly different experimental set ups such as microchemostat devices^39^.

### Statistical comparison

In order to compare the performances of *MMHelper* and *Molyso*, we manually drew ground truth detection masks in the images using the freely available GIMP drawing program and used them to quantify three parameters: the Jaccard index, precision and recall values of the automated detection (Fig. S3). We ran both software programs on our own brighfield images, and three independent sets of phase contrast images from (i) the the Locke’s laboratory^40^, (ii) the work by Sachs, *et al*. (*Molyso*)^30^ and (iii) the work by Kaiser, *et al*. (*MoMA*)^22^. In order to use *Molyso* on brightfield images, we inverted these images before analysis since the authors did not develop this software for brightfield imaging. We then directly compared the respective values for each parameter, statistical significance was tested by unpaired t test with Welch’s correction, where p ≤ 0.05 is *p ≤ 0.01 is **p ≤ 0.001 is *** and p ≤ 0.0001 is ****respectively.

## Results and Discussion

We developed *MMHelper* to work on both brightfield and phase contrast images with high detection efficiency and accuracy, this also allowing accurate extraction of data from any associated fluorescence images. In order to quantify the performances of our software, we randomly selected 5 of our brightfield datasets^28^ and analysed image time-series for 4 consecutive time-points, resulting in the analysis of 14 frames containing between 18 and 120 bacteria each. We characterised the detection efficiency as the percentage of bacteria which were detected and, from a total of 562 bacteria across all of the brightfield images, the efficiency was determined as 98 ± 1%. However, in some cases one bacterium was labelled as multiple bacteria or multiple bacteria detected as an individual bacterium. In these circumstances the detection cannot be said to be accurate, therefore we termed detection accuracy as the percentage of bacteria correctly identified by a single label and calculated it to be 80 ± 3% across the 14 previously mentioned brightfield images. Furthermore, we used *MMHelper* to analyse an image dataset acquired with a phase contrast microscope in the Locke’s laboratory^40^, obtaining a bacterial detection efficiency of 95 ± 1% and an accuracy of 65 ± 1%. This demonstrates i) the capability of *MMHelper* to detect bacteria in mother machine images in both brightfield and phase contrast modalities and ii) the capability to work equally well across independent experimental setups.

**Figure 6:**
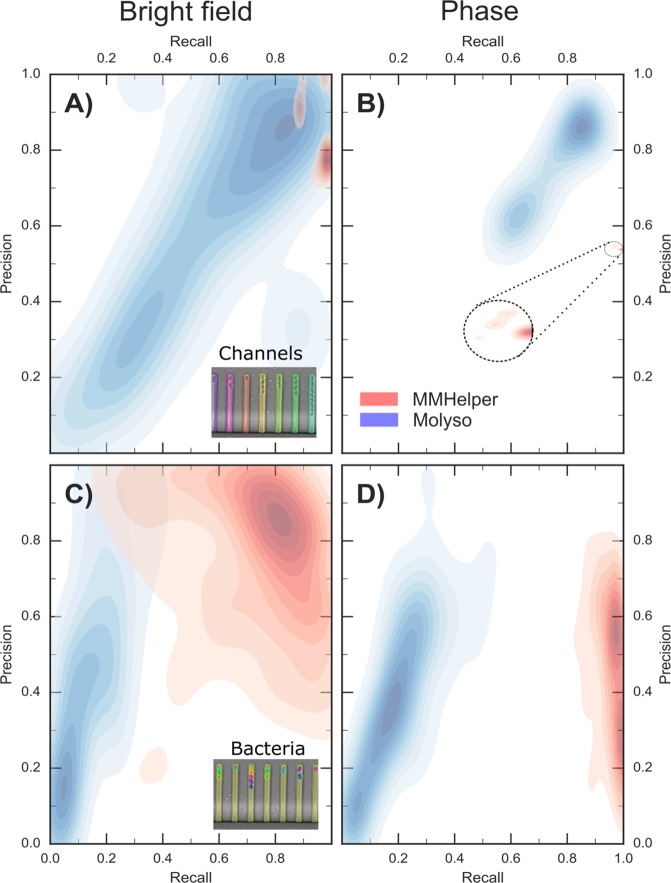
Comparison of *MMHelper* and *Molyso* performances. Kernel density estimation for precision and recall of channel detection from **(A)** brightfield and **(B)** phase contrast images via *MMHelper* (red) and *Molyso*^30^ (blue). The distribution of precision and recall values obtained via *MMHelper* on phase contrast images tightly clusters around a recall value of 1 and a precision value of 0.55. Therefore, we have zoomed this area in the dashed circle to facilitate its visualisation. **(C,D)** Corresponding kernel density estimation for precision and recall of bacteria detection. Insets: representative images of channel (A) and bacteria (C) detection.

For each software and each dataset we then measured three different parameters: detection precision as the overlap area between the detected and ground truth masks divided by the detection mask; detection recall as the overlap area divided by the ground truth mask^41,42^ (Fig. S3); and finally the Jaccard index, defined as the overlap area divided by the total combined area^42^. The use of precision and recall allows a comparison of the trade-off between ensuring no areas are missed (recall) and how precise the algorithm is, with the Jaccard index representing a combination of these values^42^. We compared these parameters for *MMHelper* and *Molyso* applied to the detection of 310 channels from our brightfield and Locke’s phase contrast images (Fig. S4 and Table 1). The corresponding Kernel Density Estimation for channel detection precision v recall is reported in Fig. 6A for brightfield and Fig. 6B for phase contrast datasets, respectively. Noteworthy, the multi-modal distribution of density for channel detection in brightfield is probably due to small variations in the quality (e.g. focus) of images acquired, resulting in the precision values varying slightly for individual images. For instance, of the total 14 birghtfield frames, the majority clustered around 0.8, one frame had a precision level of 0.9 and two frames had precision levels close to 1 (Fig. S6).

**Table 1:** Medians and median absolute deviations of Jaccard index, precision and recall for ground truth detection for *MMHelper* and *Molyso*.

	Pipeline	Bright field			Phase		
		Precision (%)	Recall (%)	Jaccard index (%)	Precision (%)	Recall (%)	Jaccard index (%)
Channels	*MMHelper*	77.8 ± 1.9	97.6 ± 1.4	77.2 ± 3.1	53.8 ± 0.2	99.4 ± 0.6	53.7 ± 0.3
	*Molyso*	64.3 ± 25.7*	58.9 ± 22.2*	42.1 ± 21.7*	79.9 ± 9.0	77.1 ± 9.4	64.6 ± 12.8
Bacteria	*MMHelper*	78.8 ± 14.6	76.3 ± 14.0	57.1 ± 14.1	47.3 ± 15.0	96.5 ± 3.5	43.9 ± 14.3
	*Molyso*	43.8 ± 21.7*	12.7 ± 7.9*	11.4 ± 6.5*	39.0 ± 17.1	19.5 ± 8.2	15.2 ± 7.1

*Molyso was not specifically developed for brightfield imaging.

As expected, according to the Jaccard index, *MMHelper* shows significantly better channel detection than *Molyso* on the brightfield datasets (p ≤ 0.0001, Fig. S4E), although *Molyso* performed better on the phase contrast dataset (p ≤ 0.0001, Fig. S4B). *MMHelper* shows a channel detection recall close to 100% for both phase contrast and brightfield images as a result of the detected channels being slightly larger than the ground truth masks, and was significantly better than *Molyso* (p ≤ 0.0001, Figs S4C and S4D). The detected channels being larger than the ground truth masks was also reflected in the precision values which were slightly lower, with *Molyso* being significantly better than *MMHelper* for phase contrast (p ≤ 0.0001, Fig. S4B), although *MMHelper* was still significantly better for brightfield (p ≤ 0.0001, Fig. S4A). This over-estimation, however, does not affect the level of accuracy of bacterial detection, see below, which is the ultimate aim of this pipeline. Figure 6C reports the Kernel Density Estimation obtained on the precision and recall values for 434 bacteria from brightfield images whereas Fig. 6D shows the Kernel Density Estimation measured for 494 bacteria from phase contrast images.

Secondly, we compared the Jaccard index of the *Molyso* and *MMHelper* performances in detecting channels from phase contrast images from the works by Sachs, *et al*. (*Molyso*)^30^ and by Kaiser, *et al*. (*MoMA*)^22^. Surprisingly, in terms of Jaccard index channel detection *MMHelper* performed better than *Molyso* on the *Molyso* image sets (p < 0.0001, Fig. S5F), whereas *Molyso* performed slightly better than *MMHelper* on the *MoMA*’s dataset (p = 0.0104, Fig. S5E). Similar to the results on our datasets, this appeared to be a result of *MMHelper* detecting slightly larger channels than the ground truth masks. *MMHelper* performed better in terms of detection recall for both *MoMA* (p ≤ 0.0001, Fig. S5C) and *Molyso* image sets (p ≤ 0.0001, Fig. S5D). Finally, *Molyso* performed better than *MMHelper* in terms of detection precision on the *Molyso* (p ≤ 0.0001, Fig. S5B) and *MoMA* image sets (p = 0.0073, Fig. S5A).

The next set of comparisons was done in terms of bacterial detection which is the ultimate goal of both *Molyso* and *MMHelper*. Therefore, ground truth masks were produced for bacteria allowing for the evaluation of bacterial detection precision, recall and Jaccard index for both *Molyso* and *MMHelper*. Bacteria detection is more difficult than channel detection, due to the inherent heterogeneity in bacterial shape and size within a clonal population. As a result, the levels of the three parameters are lower relative to channel detection (Table 1). However, according to the Jaccard index, *MMHelper* demonstrates superior performances compared to *Molyso* for both imaging modalities on our brightfield and Locke’s lab phase contrast datasets (p ≤ 0.0001, Figs S7E and S7F). In fact, *MMhelper* also performed significantly better in terms of recall (p ≤ 0.0001, Fig. S7C and Fig. S7D) and precision (p ≤ 0.0001, Fig. S7A for and p = 0.0044, Fig. S7B) on our brightfield and Locke’s lab phase contrast datasets (Table 1). We then compared the two pipelines in detecting bacteria from the *MoMA* and *Molyso* image sets. Interestingly, according to the Jaccard index, *MMHelper* again performed better than *Molyso* on the *MoMA* dataset (p = 0.0041, Fig. S8E) and their own dataset (p ≤ 0.0001, Fig. S8F). All the median values for the three parameters are listed in Table 1.

The fact that *MMHelper* outperformed *Molyso* in terms of Jaccard index for bacterial detection for all datasets further emphasises the flexibility of *MMHelper* for use on different experimental set ups as well as different bacterial species. The superior performances of *MMhelper* are probably due to the fundamental difference in the approaches to detection: the *MMhelper* algorithm is applied to the 2D images, whereas *Molyso* reduces 2D images to 1D by using line profiles and projections for channel and bacteria detection, respectively.

Finally, in order to determine the efficiency of our tracking algorithms we quantified the number of correctly tracked channels or bacteria in consecutive frames. In order to decouple tracking accuracy from detection accuracy, we excluded from the image datasets illustrated above any channels or bacteria that were incorrectly detected. *MMHelper* returned 100% and 94 ± 2% efficiency in channel and bacteria detection on the brightfield image datasets and 100% and 67 ± 4% efficiency in channel and bacteria detection on the phase contrast image datasets.

An obvious benefit of automated image analysis is the removal of human error. In order to demonstrate the superior performances of *MMHelper*, we analysed a brightfield image and the corresponding fluorescence image both via *MMHelper* and via a semi-automated approach based on ImageJ and requiring user input. Briefly, three different users measured each bacterium length from the brightfield image by drawing a straight line through the bacterium and using the corresponding intensity plot to determine where the line crossed the edges of the bacterium thus deducting the bacterial length (Fig. S9A). They then drew a box around each bacterium to measure its area (Fig. S9B). Using this same box, the fluorescence pixel intensity was extracted from the corresponding fluorescence image (Fig. S9C). For each bacterium we calculated the mean and standard deviation of these semi-automated measurements (red shaded areas in Fig. S8) and compared these values to the ones obtained via *MMHelper* (blue circles in Fig. S9). Whereas *MMHelper* is able to accurately detect the bacterial contour, the semi-automated approach consistently overestimates the area of individual bacteria and underestimates the GFP fluorescence from single bacteria. Therefore, in order to allow a direct comparison between the values obtained via the two approaches, Fig. S4 reports each single-bacterium value normalised to the corresponding mean of all the single-bacterium values. This allows us to demonstrate that *MMHelper* robustly and accurately extracts single-cell data with 69% of *MMHelper* measurements falling within 1 S.D. of the mean, 97% within 2 S.D., and 100% within 3 S.D. of the mean of the values obtained via the semi-automated approach (Fig. S9C).

Input images can vary in quality and magnification and the bacterial geometry can vary depending on species, phase of growth, and due to the phenotypic heterogeneity inherent in clonal populations. In order to account some of these variations, some of the input parameters for *MMHelper* can be varied accordingly. For example, tuning the scale factor accounts for changes in image magnification. Furthermore, we are also developing a graphic interface for the manual correction of *MMHelper* output, where needed, which aims to make this process both easier and more efficient.

*MMHelper*, to the best of our knowledge, is the only automated analysis pipeline that has been designed for the analysis of both brightfield and phase contrast images acquired with the mother machine. Some researchers use fluorescent tags in order to perform their image analysis^24,29^, but this requires exposure to strong light sources that are known to be extrinsic damage-producing agents^43^. Conversely, *MMHelper* allows the extraction of single-bacterium length and area measurements from brightfield or phase contrast images, allowing measurements of single-cell parameters such as growth rate and elongation time that are crucial when investigating phenomena such as ageing^19,44,45^, bacterial susceptibility^28,46^ and cell size regulation^20^.

When needed, fluorescence can be used as a reporter for intracellular pH, gene expression, or substrate accumulation. Therefore, *MMHelper*, will facilitate the study of mutagenesis, gene regulation, and cellular homeostasis at the single cell level. Furthermore, when current microbiological assays are performed at the population level, viable but non-culturable bacteria are overlooked. VBNC cells are a subpopulation of cells which enter a dormant state allowing them to survive otherwise lethal concentrations of antibiotics but they do not resuscitate immediately upon exposure to fresh media^47^. As a result, they can be responsible for the recalcitrance of chronic infections and act as a stepping stone in the development of antibiotic resistance^47^. In contrast, our high-throughput system can be used to ensure that non-growing phenotypes can be detected for example during the testing of new antimicrobials or exposure to stress. In this respect, we have recently used *MMHelper* to demonstrate that persister and viable but non culturable *E*. *coli* cells differentially regulate genes associated with tryptophan metabolism before exposure to ampicillin^28^ opening new opportunities to map the detailed biochemical makeup of these clonal subpopulations.

## Conclusion

*MMHelper* provides an automated framework for the analysis of any type of microscopy images acquired with the mother machine. This automated approach provides large amounts of data with a high level of accuracy in both a time efficient and reproducible manner. For instance, on average it would take a user approximately an hour to analyse a series of 8 consecutive images using ImageJ, whereas *MMHelper* can acquire the same information in approximately one minute, requiring only a limited amount of manual editing of the output data thanks to the high level of accuracy provided. After thoroughly testing *MMHelper* to analyse our own mother machine experiments performed on different experimental set-ups and different bacterial strains we are now making this open-source software available for all the research groups already using the mother machine around the world. Finally, we believe that, thanks to the ease of installation and use, *MMHelper* will be an incentive for researchers from a variety of scientific backgrounds to employ this powerful technology for investigating biological questions with single cell resolution.

## Supplementary information

Supplementary materials
