# Supplementary material for: MMHelper: An automated framework for the analysis of microscopy images acquired with the mother machine
Source: Sci Rep. 2019 Jul 12;9:10123. doi: 10.1038/s41598-019-46567-0 (PMC6626022; doi:10.1038/s41598-019-46567-0)
Supplement: Supplementary file 1 — Supplementary materials [file 41598_2019_46567_MOESM1_ESM.pdf]

Supplementary Material

MMHelper: An automated framework for the analysis of microscopy images acquired with the mother machine

Ashley Smith[1,2†], Jeremy Metz[1,2†*], Stefano Pagliara[1,2*]
[1]Living Systems Institute, University of Exeter, Exeter, United Kingdom
[2] Biosciences, University of Exeter, Exeter, United Kingdom

[†]These authors contributed equally to the work
*** Correspondence:**
Stefano Pagliara
s.pagliara@exeter.ac.uk
Jeremy Metz
j.metz@exeter.ac.uk

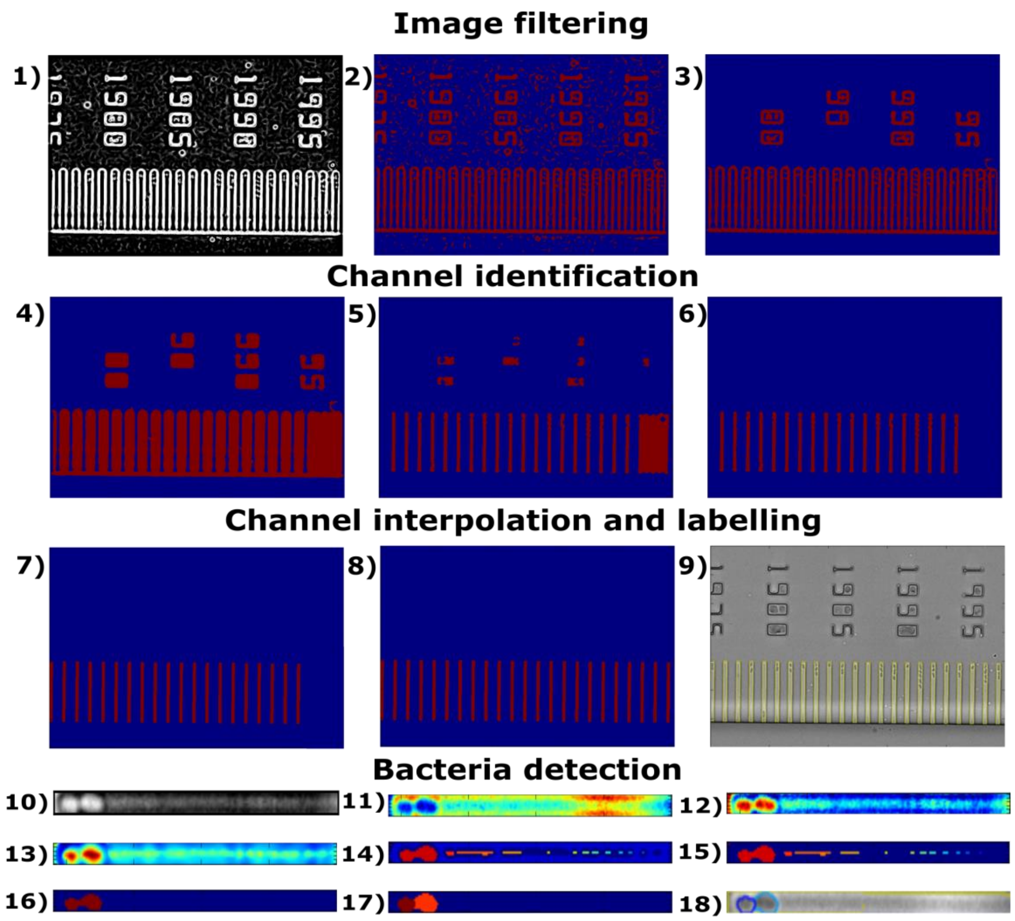

Fig. S1. Overview of the detection pipeline. The image is filtered (1) using Sobel (phase contrast) or Frangi (bright field). This is followed by thresholding (2) using Li's minimum cross entropy threshold, before finally being filtered based on size (3). The size filtered image is dilated and the gaps (channel centres filled (4). The size filtered image is then subtracted to leave only the centre of the channels (5). The channel centres are then extrapolated and only "good" channels are kept (6). An "average" shape of a channel is determined and used for each of the detected channels (7). The spacing is then determined and any missing channels "stamped" in to place (8). This final mask can then be used to extract the well profiles for bacteria detection. The final detected wells can be seen as yellow contours (9). For display purposes, images 10-18 have been rotated 90°. If the original image was acquired in bright field then it must be inverted (10). Then, in order to account for any variation in background intensity (11), the background is subtracted using a rolling ball filter (12) and then scale-space filtered (13). Using Li's minimum cross entropy based algorithm on all the channels within an image, a threshold is determined. This threshold is applied to each well individually in order to identify markers (14) for watershed segmentation (15). The result of the watershed segmentation is then size filtered (16), before a final "splittling" algorithm is applied to their skeleton based on a combination of distance transformation and pixel intensity (17). The contours of the final labels are then plotted on the original image for visualisation purposes (18).

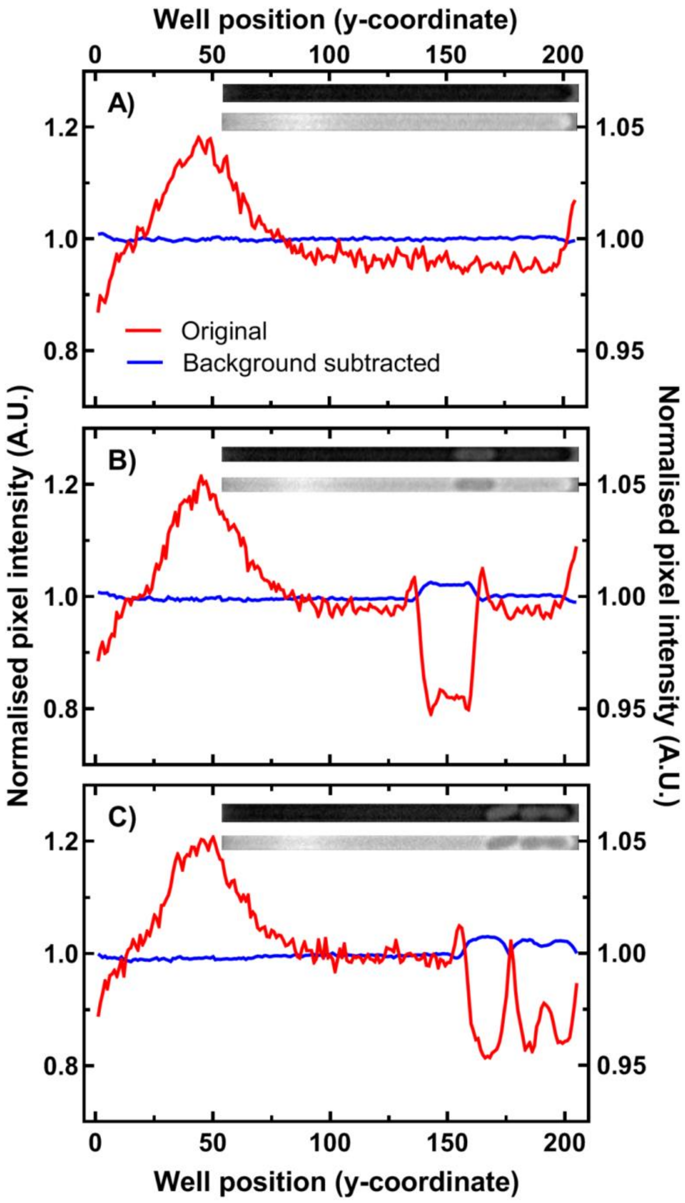

Figure S2. Comparison of channel profiles before and after background subtraction. The MMHelper pipeline uses a rolling ball filter to normalise the background profile (blue lines) to account for the changing background along the original channel profile (red lines). We measured the profile from the channel entrance (0μm) to the end of the channel (23.5μm) for a channel containing no bacteria (A), a channel with a single bacterium around the centre of the channel (B) and for a channel with multiple bacteria at one end (C) and plotted the relative intensity at each position within the channel. In all cases, the rolling ball filter removed the variation associated with the original image. It is important to note that the images were inverted prior to background subtraction, which is why we see an increase in intensity for the normalised (blue line) bacteria compared to the original (red line).

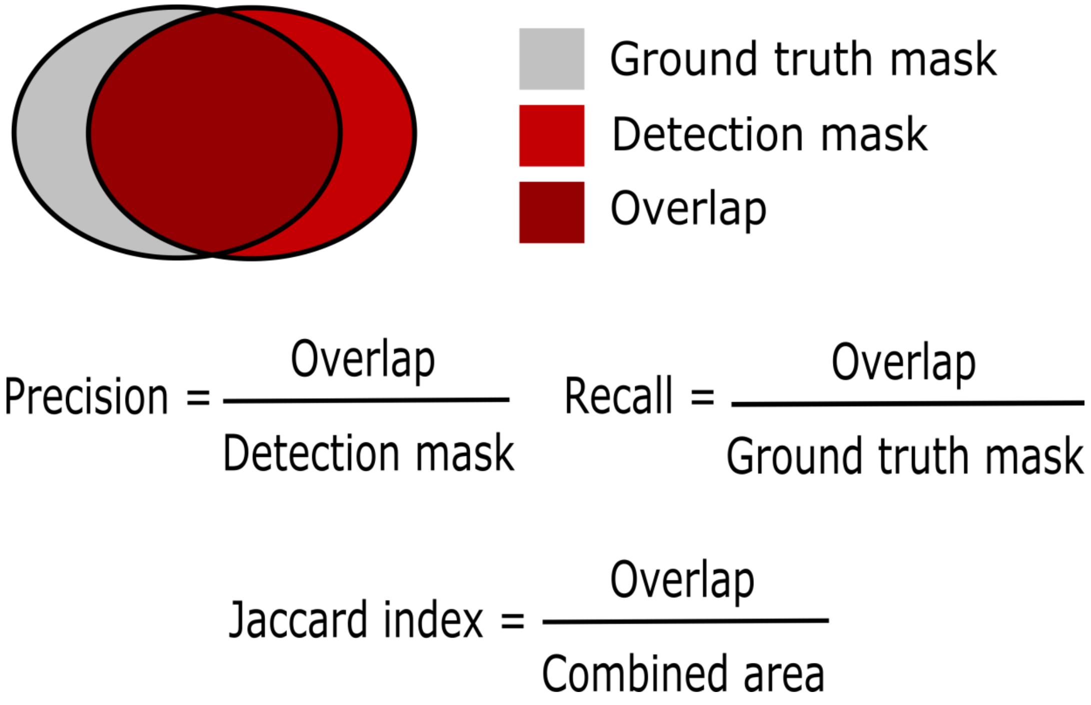

Figure S3. Schematic illustrating our definition of detection precision and recall. Ground truth masks were drawn manually using the software GIMP and detected masks were produced from the automatic analysis pipelines. The precision value is termed as the percentage overlap area between the ground truth and detected mask divided by the total area of the detected mask. The recall value is the percentage overlap divided by the total area of the ground truth mask. Finally, the Jaccard index is determined as the overlap area divided by the total combined area.

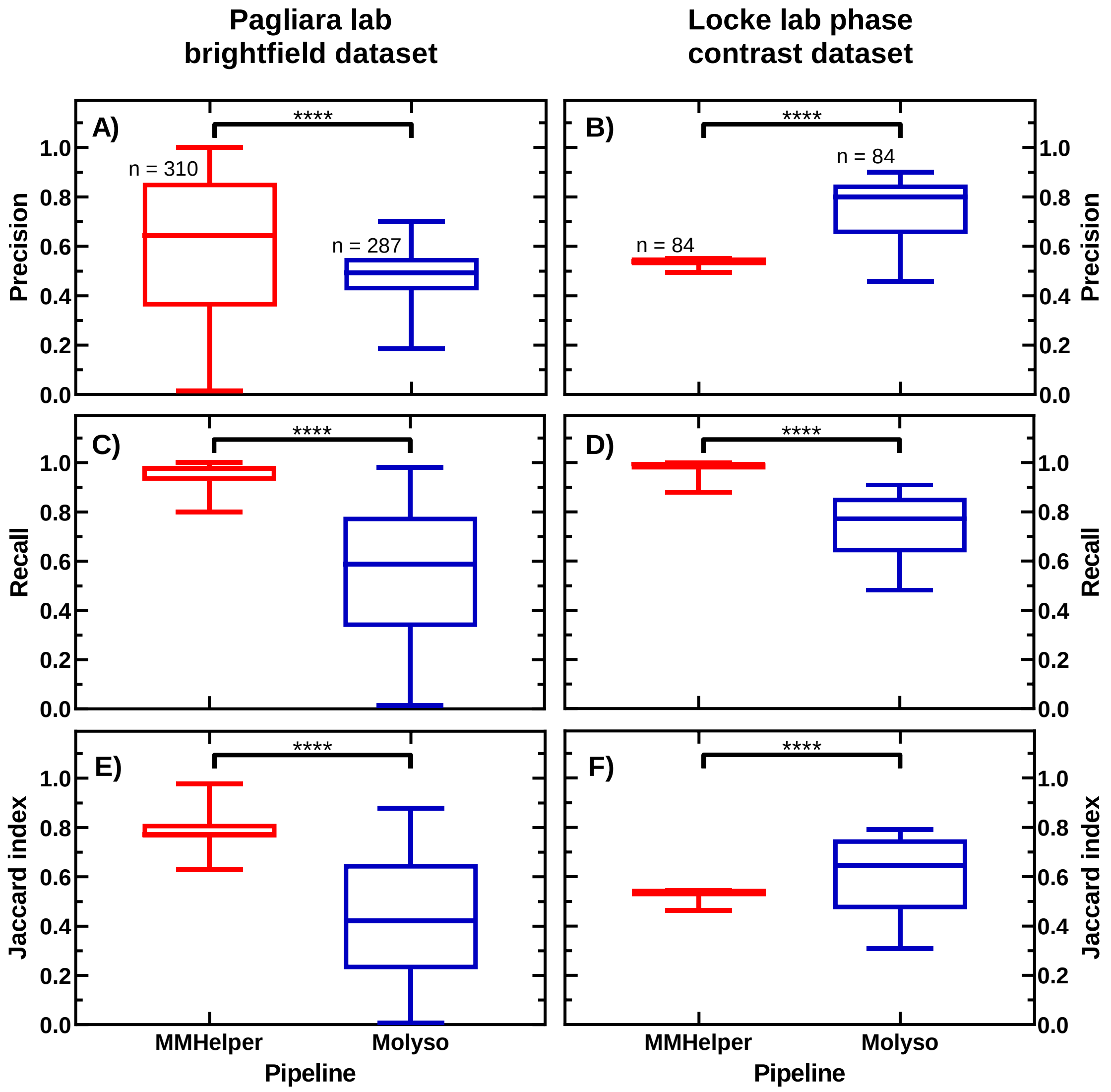

Figure S4. Comparison of channel detection in our brightfield and phase contrast datasets via MMHelper and Molyso in terms of Jaccard index, precision and recall. Using the ground truth masks manually drawn using GIMP, we determined the levels of precision and recall for the detected channels from *MMHelper* and *Molyso*, respectively. The results show that the median precision levels of *MMHelper* are approximately 0.8 for **(A)** brightfield and 0.55 % for **(B)** phase contrast images because the detected channels are consistently larger than the ground truth masks. This is reflected by the high levels of recall in both **(C)** brightfield and **(D)** phase contrast images. *Molyso*, in comparison, only out performs *MMHelper* on phase contrast precision. Finally, the Jaccard index shows that *MMHelper* was significantly better on **(E)** brightfield, but *Molyso* was significantly better for our **(F)** phase contrast dataset. Statistical significance was determined using a Welch's correlation as described in the methods section.

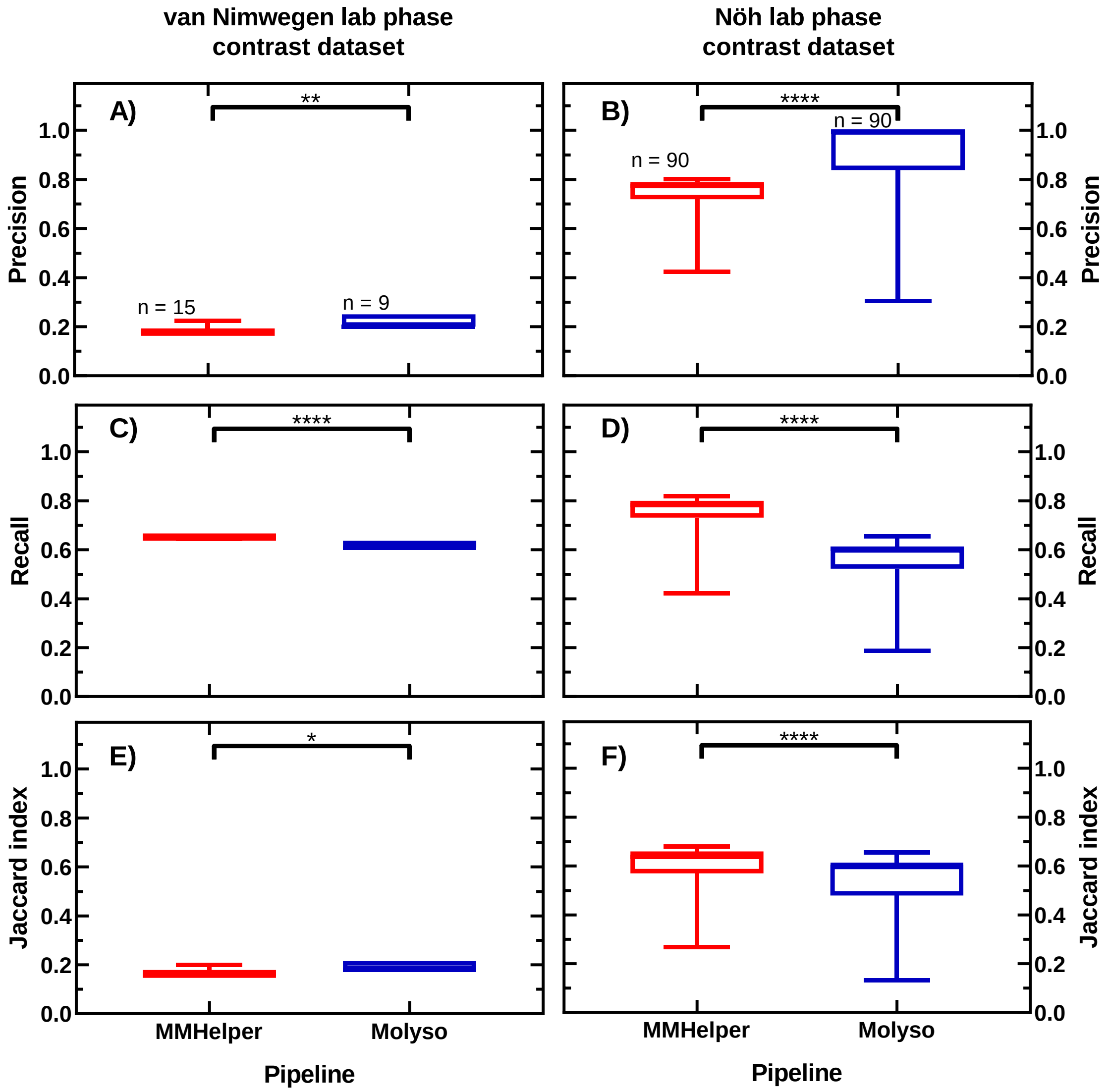

Figure S5. Comparison of channel detection in both *MoMA* and *Molyso* example phase contrast datasets via MMHelper and Molyso in terms of Jaccard Index, precision and recall. The **(A)** precision and **(C)** recall values were calculated for the *MoMA* example dataset. Similar to the **(B)** precision and **(D)** recall for *Molyso*'s dataset, *MMHelper* showed significantly better recall due to slightly larger detected channels compared to the ground truth masks, whereas *Molyso* was significantly better in terms of precision. The Jaccard index, determined by dividing the overlap of an area by the total combined area, was calculated for the channels detected in the **(E)** *MoMA* and **(F)** *Molyso* datasets, respectively. Interestingly, *Molyso* was slightly better for the *MoMA* dataset, but *MMHelper* performed significantly better on *Molyso*'s own dataset. Statistical significance was determined using a Welch's correlation as described in the methods section.

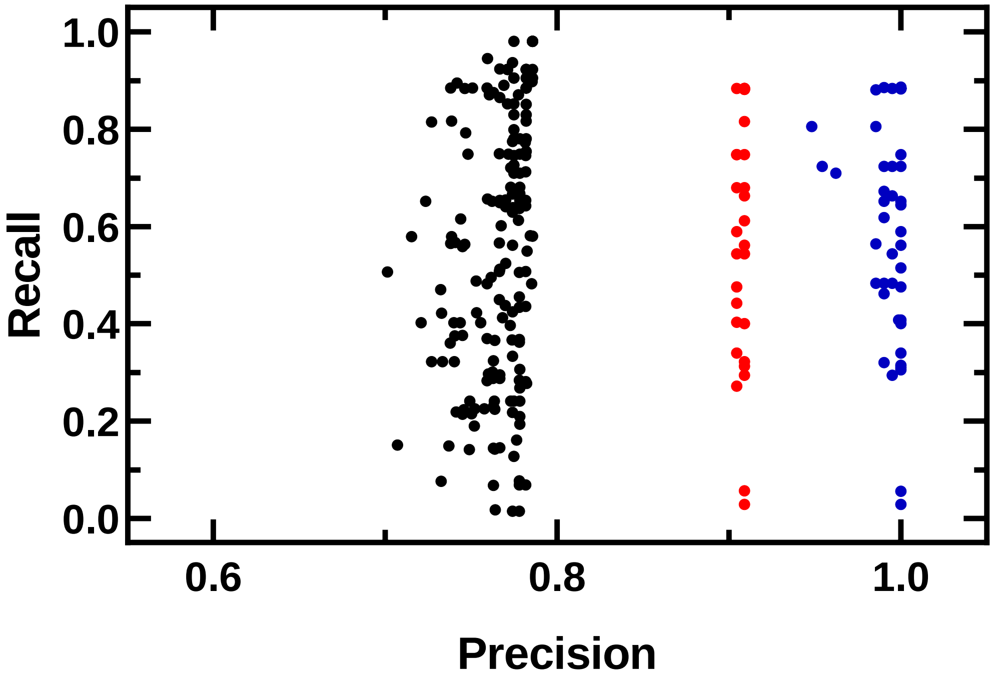

Figure S6. Scatter plot of precision and recall values for channel detection. Scatter plot of representative precision and recall values for channel detection across all 14 bright field frames. The plot clearly shows 3 different clusters of data; the majority of frames across all experiments had a precision level around 0.8, one frame had a precision level around 0.9 and two frames had a precision level around 1.0. This grouping results in the multimodal distribution in the kernel density plots.

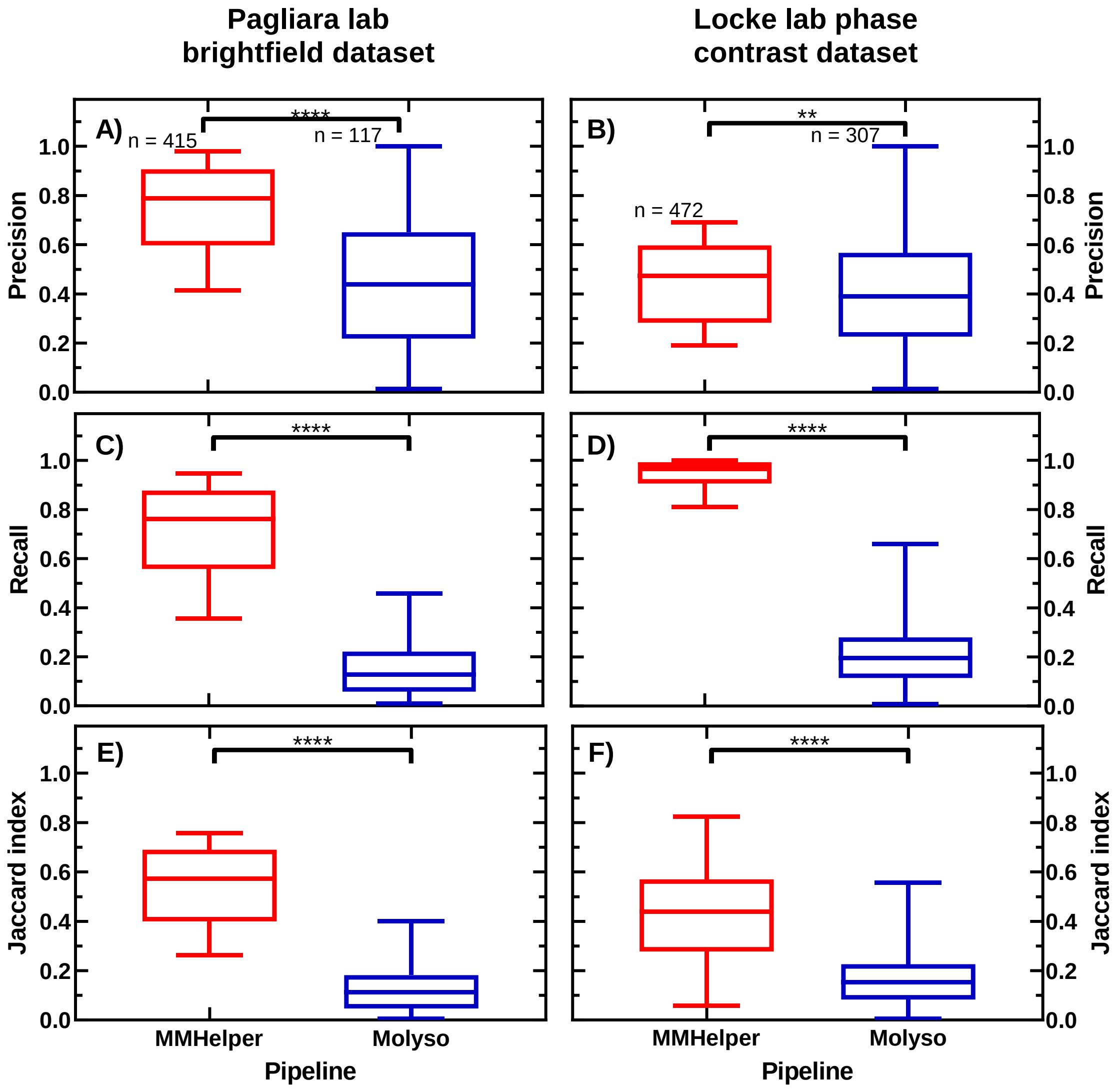

Figure S7. Comparison of bacterial detection in our brightfield and phase contrast datasets via MMHelper and Molyso in terms of Jaccard index, precision and recall. Using the ground truth masks manually drawn using GIMP, we determined the levels of precision and recall for the detected channels from *MMHelper* and *Molyso*, respectively. The results show that *MMHelper* had significantly higher precision levelsfor **(A)** brightfield and **(B)** phase contrast images. Similarly, *MMHelper* had significantly higher levels of recall in both **(C)** brightfield and **(D)** phase contrast images. As a result, the Jaccard index shows that *MMHelper* was again significantly better on **(E)** bright field and **(F)** phase contrast dataset. Statistical significance was determined using a Welch's correlation as described in the methods section.

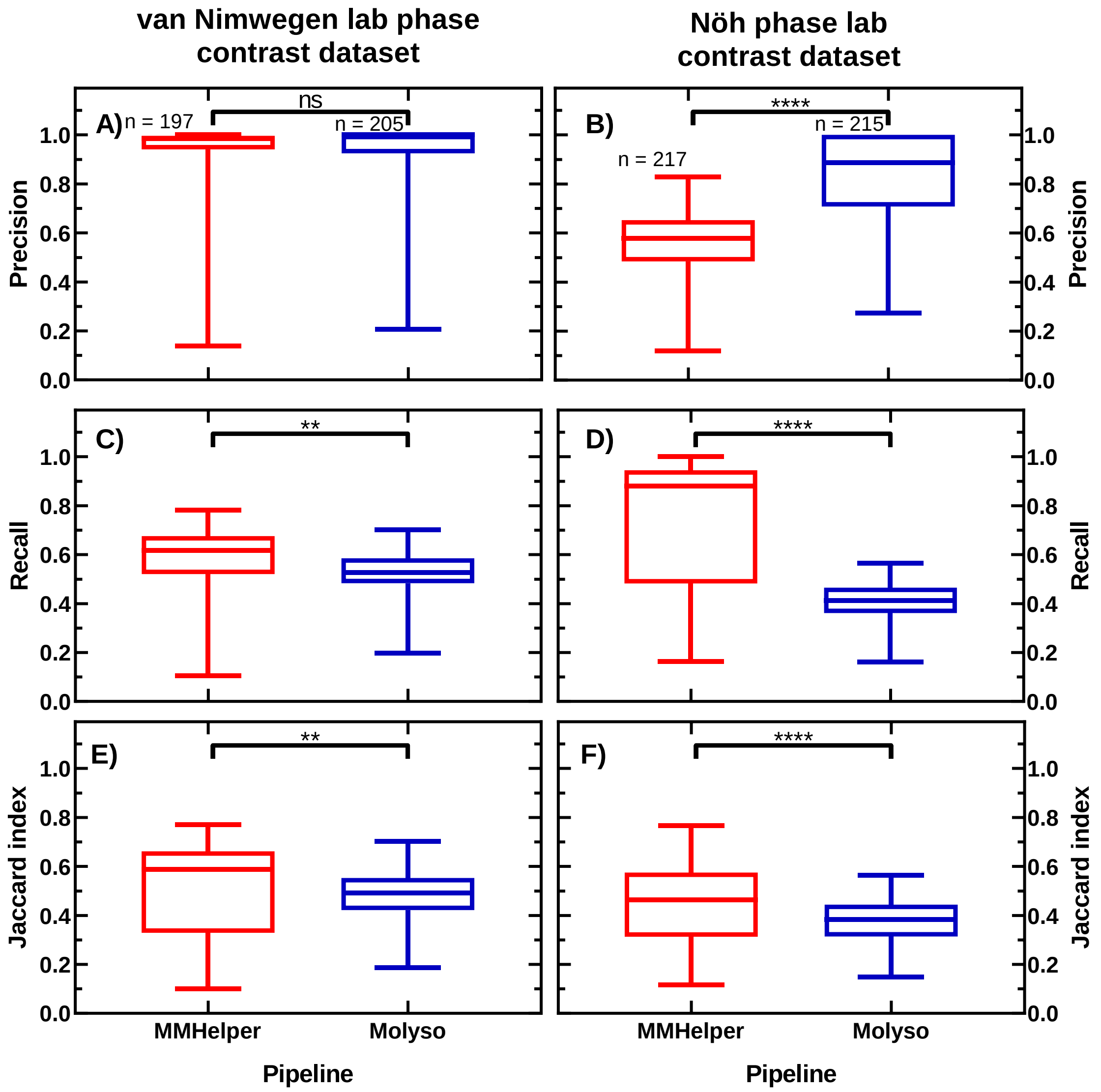

Figure S8. Comparison of bacterial detection in both *MoMA* and *Molyso* example phase contrast datasets via MMHelper and Molyso in terms of Jaccard Index, precision and recall. There was no significant difference between the **(A)** precision values on the *MoMA* dataset. However, *MMHelper* performed slightly better on **(C)** recall and therefore also with respect **(E)** Jaccard index. Interestingly, although *Molyso* was significantly better on its own **(B)** precision values, *MMHelper* outperformed it on **(D)** recall and **(F)** Jaccard index. Statistical significance was determined using a Welch's correlation as described in the methods section.

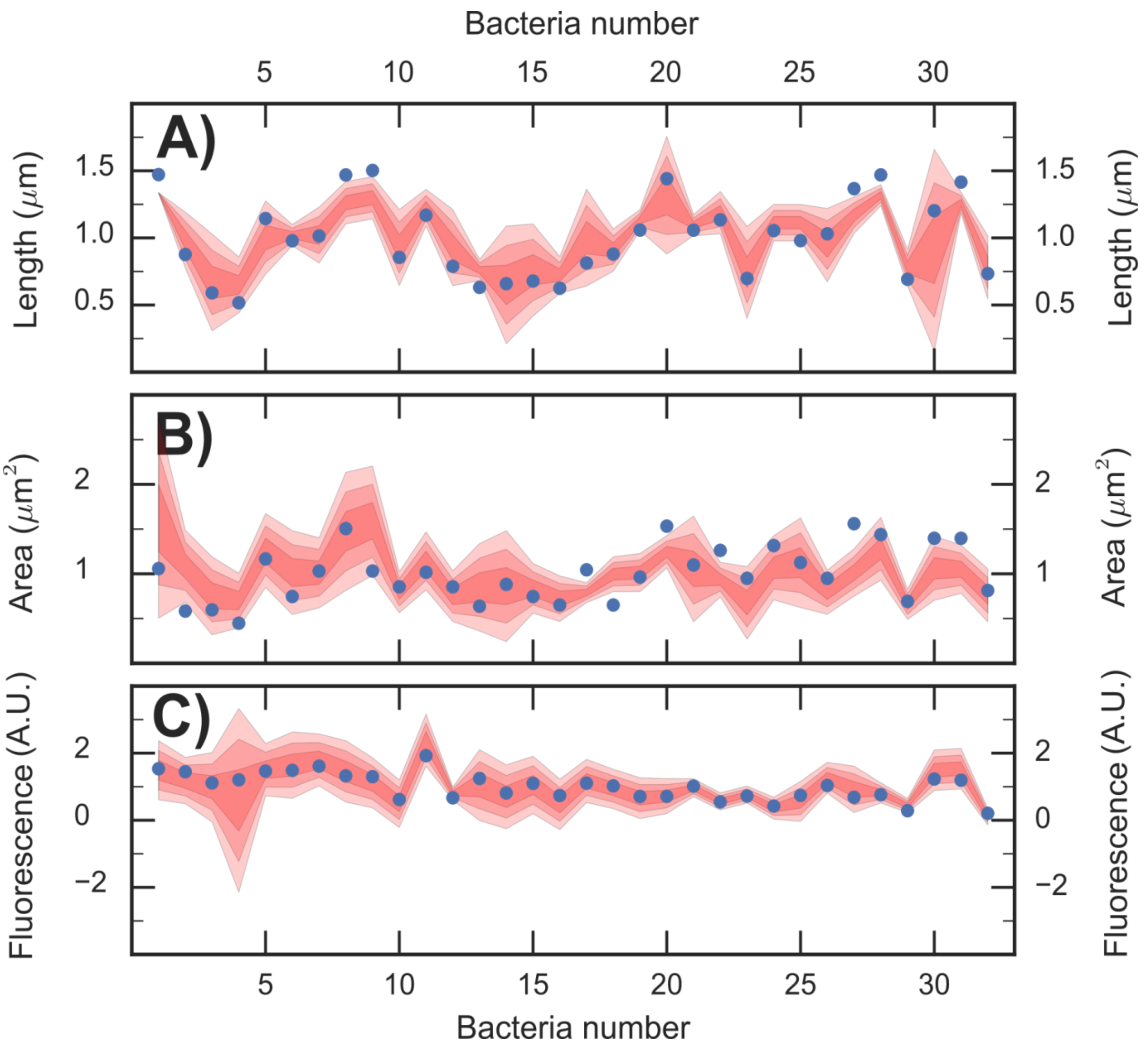

Figure S9. Comparison of single bacterium parameters obtained via *MMHelper* and a semi-automated approach requiring user input. (A) Bacterial length, **(B)** area, and **(C)** GFP fluorescence measured via *MMHelper* (blue dots) and a semi-automated method based on ImageJ and performed by three different users, the red bands indicate the mean ± 1 S.D. (darkest red band), mean ± 2 S.D., and mean ± 3 S.D. (lightest red band), respectively. To account for the consistently more precise automated measurements, both the manual and automatic measurements were normalised by dividing the measurement for each bacterium by the mean of all the single-bacterium measurements.
